# Metabolic and molecular mechanisms of spine color formation in Chinese red chestnut

**DOI:** 10.3389/fpls.2024.1377899

**Published:** 2024-05-21

**Authors:** Qian Qiao, Yun Gao, Qingzhong Liu

**Affiliations:** ^1^ Shandong Key Laboratory of Fruit Biotechnology Breeding, Shandong Institute of Pomology, Taian, Shandong, China; ^2^ College of Plant Protection, Shandong Agricultural University, Taian, Shandong, China

**Keywords:** Chinese chestnut, red chestnut, spine, transcriptome, anthocyanin, cyanidin

## Abstract

The spines of Chinese red chestnut are red and the depth of their color gradually increases with maturity. To identify the anthocyanin types and synthesis pathways in red chestnut and to identify the key genes regulating the anthocyanin biosynthesis pathway, we obtained and analyzed the transcriptome and anthocyanin metabolism of red chestnut and its control variety with green spines at 3 different periods. GO and KEGG analyses revealed that photosynthesis was more highly enriched in green spines compared with red spines, while processes related to defense and metabolism regulation were more highly enriched in red spines. The analysis showed that the change in spine color promoted photoprotection in red chestnut, especially at the early growth stage, which resulted in the accumulation of differentially expressed genes involved in the defense metabolic pathway. The metabolome results revealed 6 anthocyanins in red spines. Moreover, red spines exhibited high levels of cyanidin, peonidin and pelargonidin and low levels of delphinidin, petunidin and malvidin. Compared with those in the control group, the levels of cyanidin, peonidin, pelargonidin and malvidin in red spines were significantly increased, indicating that the cyanidin and pelargonidin pathways were enriched in the synthesis of anthocyanins in red spines, whereas the delphinidin pathways were inhibited and mostly transformed into malvidin. During the process of flower pigment synthesis, the expression of the *CHS*, *CHI*, *F3H*, *CYP75A*, *CYP75B1*, *DFR* and *ANS* genes clearly increased, that of *CYP73A* decreased obviously, and that of *PAL*, *4CL* and *LAR* both increased and decreased. Notably, the findings revealed that the synthesized anthocyanin can be converted into anthocyanidin or epicatechin. In red spines, the upregulation of *BZ1* gene expression increases the corresponding anthocyanidin content, and the upregulation of the *ANR* gene also promotes the conversion of anthocyanin to epicatechin. The transcription factors involved in color formation included 4 *WRKYs*.

## 1 Introduction

The *Castanea* Mill plant genus is native to the northern temperate regions of Asia, Europe, Africa and the Americas, with approximately 10 species. Among them, Chinese chestnut (*C. mollissima* Blume), European chestnut (*C. sativa* Mill), Japanese chestnut (*C. crenata* Sieb. et Zucc), and American chestnut (*C. dentata* Borkh) are the main edible chestnuts (Wang et al., 2022a) and play important roles in the fight against hunger (Beccaro et al., 2019; Wang et al., 2020a), and Chinese chestnut is a famous dried fruit and woody grain that is not only the most widely cultivated in the world but also the most productive. Originating in China, Chinese chestnut has a long cultivation history and a vast distribution area. As early as 6,000 years ago, ancient people were eating wild chestnut. After long-term selective breeding by humans, today’s chestnut has the characteristics of being exposed to sunlight and having a developed root system, strong adaptability, good nut quality and strong stress resistance. Moreover, the nut tastes delicious and is highly valuable in nutrition and medicine (Zhang et al., 2022). Chinese chestnut has become an important economic forest tree in China, occupies an important position among the world’s edible chestnuts, and has become an important edible chestnut germplasm resource (Liu et al., 2015). Chestnut is widely distributed in China; from Liaoning and Jilin in the north to Guangdong and Guangxi in the south, there are 26 provinces and cities with chestnut cultivation and distribution. The latitude span from north to south reaches 23°, and includes the subcold zone, the warm zone, the north subtropical zone and the subtropical zone. In terms of regional distribution, six subspecies groups have formed in North China, the middle and lower reaches of the Yangtze River, Northwest China, Southeast China, Southwest China and Northeast China (Zhang et al., 2020b). The Chinese chestnut resources are also very rich and more than 350 chestnut varieties have been discovered and identified (Lan et al., 2010). These include not only rare resources such as chestnut, red chestnut, hanging chestnut, and three-season chestnut but also undomesticated resources such as wild chestnut that are widely distributed. Among these resources, red chestnut was discovered in the 1950s during a field investigation by researchers at the Shandong Institute of Pomology. Its new leaves in spring and the entire growing season of the spines are red, and the spines gradually turn deeper red with maturity. Hybridization tests have proven that the mutation responsible for its red coloration is a reverse mutation of the gene, but the mutation site and its color formation mechanism are unclear.

The accumulation of anthocyanins affects color formation in branches, leaves, buds, flowers, fruits and other organs. In general, pelargonidin and cyanidin provide red pigments for flowers and fruits, and peonidin contributes greatly a large contribution to the purplish red color of plant tissues, whereas delphinidin, petunidin and malvidin are responsible for the bluish purple and purplish red colors of plants (Jaakola, 2013; Khoo et al., 2017; Wang et al., 2022b). However, the relationship between anthocyanin accumulation and the plant organ coloration varies among different species. Anthocyanin coloration can be consistent among different plant species. For example, cyanidins are the main anthocyanins responsible for the pink and red petals of *Camellia japonica* and *Prunus persica* (Cheng et al., 2014; Fu et al., 2021). However, anthocyanin coloration can also show species specificity. For instance, delphinidins are the dominant anthocyanins in most plants with pure blue petals, whereas the petals of transgenic rose hybrids with a high percentage of delphinidins (up to 95%) are not as pure blue as those of other plants (Katsumoto et al., 2007).

The biosynthesis of anthocyanins mainly involves structural genes and regulatory genes (Wang et al., 2020b; Yan et al., 2021). Structural genes, such as *PAL*, *C4H*, *4CL*, *CHS*, *CHI*, *F3H*, *F3’H*, *F3’5’H*, *FLS*, *DFR*, *ANS*, *ANR*, *UFGT* and other genes, are involved mainly in the phenylpropanoid biosynthesis, flavonoid biosynthesis and anthocyanin synthesis pathways (Pelletier et al., 1999; Wang et al., 2023). Differential expression of structural genes affects the type and content of anthocyanins, which affects the color of the plants. For example, *ANS* is only expressed in sepals of *Forsythia intermedia*, but not in antheral petals (Rosatic et al., 1999). The production of anthocyanins in purple pepper is mainly caused by high expression of downstream genes such as *DFR*, *ANS* and *UFGT* (Tang et al., 2020). The accumulation of cyanidin and delphinidin in colored cotton fiber is closely related to the *F3’H*, *F3’5’H* and *DFR* gens. The expression abundance of *F3’H* and *F3’5’H* is an important factor affecting color. A high level of *F3’5’H* and a low level of *F3’H* can effectively synthesize blue flowers dominated by delphinidin (Feng et al., 2014). Regulatory genes mainly refer to transcription factors that can regulate the transcription of structural genes. The transcription factors involved in regulating anthocyanin biosynthesis mainly include *MYB*, *bHLH* and *WD40*. The MYB proteins regulated by these three transcription factors are composed of a series of incomplete R repeat sequences that can specifically bind to DNA double strands (Yan et al., 2021). It can both promote anthocyanin synthesis and inhibit anthocyanin synthesis. The MYB protein with a promoting effect is mainly R2R3-Myb, and the inhibitory MYB proteins include some R2R3-Myb and R3-Myb proteins (Li et al., 2019; Wang et al., 2019). In addition, environmental factors also have important impacts on MYB expression (Jun et al., 2015). The MBW-TF ternary protein complex of MYB-bHLH-WD40 controls the MBW complex and the downstream accumulation of anthocyanins (Li et al., 2020; Yang et al., 2020).

Therefore, the identification of anthocyanin types and synthesis pathways in red chestnut and the identification of key structural genes or transcription factors that regulate anthocyanin biosynthesis pathways would help explain the cause of red spines, and lay a foundation for the generation of red mutants and the analysis of the underlying genetic mechanisms. In this study, a combination of transcriptome and anthocyanin metabolism data was used to further analyze the sources of differences and identify key differentially expressed genes (DEGs) sites to provide molecular biological evidence for the generation and inheritance of the red chestnut mutation.

## 2 Materials and methods

### 2.1 Materials

**Figure 1:**
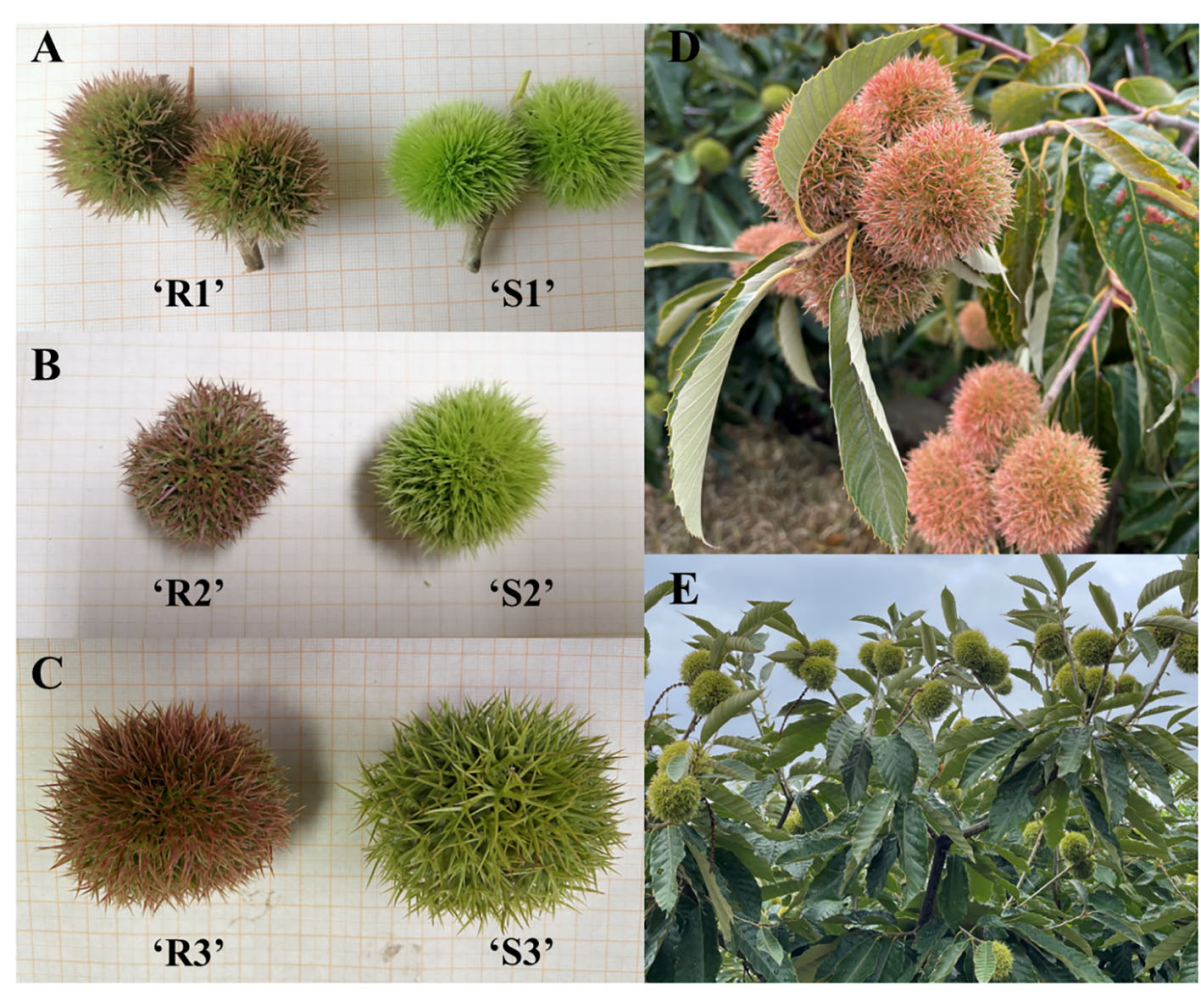
Test materials. **(A)** Sampling diagram on July 5; **(B)** Sampling diagram on August 5; **(C)** Sampling diagram on September 5; **(D)** Chinese red chestnut at the fully ripe fruit stage; **(E)** ‘Song jiazao’ at the fully ripe fruiting stage.

The spines of Chinese red chestnut (with red spines) (R) and ‘Songjiazao’ (with green spines) (S) at 3 developmental stages were used as the experimental materials (**Figure 1**) and were named R1, R2, R3, S1, S2, and S3. ‘Songjiazao’, a new early-maturing variety bred by our research group, served as the control variety. This verity is produced in the village of Songjiazhuang, Mata Town, Daiyue District, Tai’an City, Shandong Province, and its spines are always green during the development period. Samples were collected at three time points (07.05, 08.05, 09.05, and 2022) from Songjiazhuang village, Mata town, Daiyue District, Tai’an City, Shandong Province. The samples were frozen with liquid nitrogen immediately after collection and then stored in an ultralow temperature (-80 °C) refrigerator.

### 2.2 Transcriptome sequencing and analysis

Total RNA was extracted from the samples using the TRIzol (Invitrogen) method, and genomic DNA was removed using DNase I (TaKaRa). The quality of the RNA samples was measured by a 2100 Bioanalyzer (Agilent) and ND-2000 (NanoDrop Technologies) to ensure sufficient quality for the transcriptome sequencing of the combined samples (OD260/280 = 1.8~2.2, OD260/230≥2.0, RIN≥6.5, 28S:18S≥1.0, >1 μg). The RNA library was constructed using a TruSeq™ RNA sample preparation kit (Illumina, San Diego, CA, USA). Though A-T base pairing with polyA using magnetic beads with oligo (dT), mRNA was isolated from total RNA for analysis of transcriptome information. The Chinese red chestnut genome assembled by our research group was used as the reference genome for database analysis (https://figshare.com/articles/dataset/Genome_annotation_of_Hongli_Castanea_mollissima_Blume_/21614721/1). The cDNA libraries were sequenced via paired-end sequencing using the Illumina NovaSeq 6000 System platform (Illumina, CA, USA). We used DESeq2 (Michael et al., 2014) software to test the statistical enrichment of DEGs, which were identified as those with a fold change ≥2 and a false discovery rate (FDR) < 0.05, in GO terms and KEGG pathways.

### 2.3 qRT-PCR validation

Twenty DEGs related to anthocyanin metabolism (**Supplementary Table S1**) were selected from the transcriptome sequencing results, and *Actin* was used as the internal reference gene (Chen et al., 2019a). The relative gene expression levels were calculated using the 2^-△△t^ method. According to the results, the transcriptome was compared with the real-time fluorescence quantitative results, and the log_2_fold change was visualized in a longitudinal histogram to analyze whether the expression trend of the transcriptome results was consistent with the qRT-PCR verification results.

### 2.4 Anthocyanin species and content determination

The anthocyanin metabolomics was analyzed by UPLC-MS/MS at Wuhan Meiwei Metabolism Company. The sample extraction process was as follows: A ball mill (30 Hz, 1.5 min) was used to grind the sample after vacuum freeze-drying to obtain powder, and 50 mg of the resulting powder was weighed and dissolved in 500 μL of extraction solution (50% methanol aqueous solution containing 0.1% hydrochloric acid), swirled for 5 min, ultrasonicated for 5 min, and centrifuged for 3 min (12,000 r/min, 4°C). The supernatant was combined twice, and the sample was filtered through a microporous filter membrane (pore size of 0.22 μm) and stored in a sample vial for LC-MS analysis.

The data acquisition instrument system mainly included an ultra-performance liquid chromatograph, (UPLC, ExionLC™ AD, https://sciex.com.cn/) and a tandem mass spectrometer, (MS/MS, QTRAP^®^ 6500+, https://sciex.com.cn/). The chromatographic conditions were as follows: column ACQUITY BEH C18 1.7 µm, 2.1 mm * 100 mm; mobile phase A was ultrapure water (0.1% formic acid added), and phase B was methanol (0.1% formic acid added). The elution gradient was as follows: the mobile phase was 5% B at 0.00 min, increased to 50% at 6.00 min, increased to 95% at 12.00 min, maintained at 95% for 2 min, decreased to 5% at 14 min, and balanced for 2 min. The flow rate was 0.35 mL/min, the column temperature was 40°C, and the sample size was 2 μL (Chen et al., 2018). The mass spectrum conditions mainly included an electrospray ionization (ESI) temperature of 550°C, a mass spectrum voltage of 5500 V in positive ion mode, and a curtain gas (CUR)- pressure of 35 psi.

MetWare database (MWDB) analysis was performed based on standard products, and a qualitative analysis of mass spectrometry data was conducted. Quantification was performed via multiple reaction monitoring (MRM) analysis by triple four-bar mass spectrometry. The anthocyanin levels were detected by MetWare (http://www.metware.cn/) based on the AB Sciex QTRAP 6500 LC-MS/MS platform.

The fold change obtained from the univariate analysis and the variable importance in projection (VIP, which is the importance of the effect of the difference between corresponding metabolites in the classification and discrimination of samples of each group in the model) of the OPLS-DA model were combined to identify the differentially abundant metabolites among the samples. The screening criteria for metabolites were the following: fold change ≥2, fold change ≤0.5, and VIP ≥1. The selected differentially abundant metabolites were annotated based on the KEGG database (Kanehisa and Goto, 2000).

## 3 Results

### 3.1 RNA-seq and assembly analyses

#### 3.1.1 Quality detection and analysis of RNA samples from transcriptome sequencing

**Figure 2:**
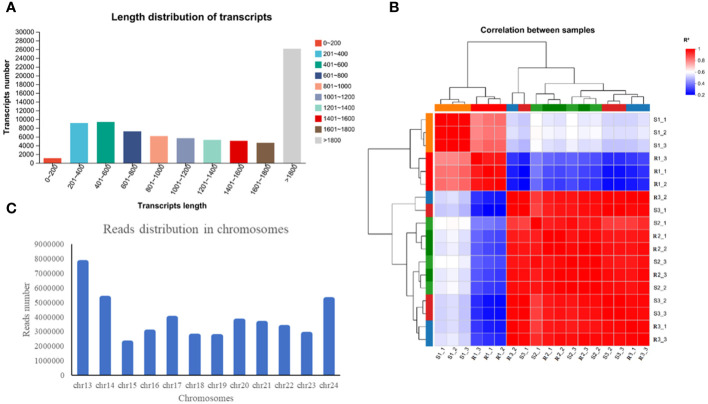
Detection and analysis of the transcriptome quality. **(A)** Length distribution of transcripts. **(B)** Read distribution in chromosomes. **(C)** Read distribution in chromosomes.

In this study, a total of 119.34 Gb of clean data were obtained, and more than 6.07 Gb of clean data were obtained from all the samples. In the data, the percentage of Q20 bases was greater than 97.06% and the percentage of Q30 bases was greater than 91.94%. The GC content ranged from 44.14% to 45.24% (**Supplementary Table S2**). The number of transcripts with a length greater than 1800 bp was 26,123, accounting for 32.81% of the transcripts; the remaining transcripts were concentrated at approximately 200 and 1000 bp, accounting for 31,833 and 39.98% of the total number of transcripts, respectively (**Figure 2A**). According to the FPKM values of gene expression, correlation coefficients were calculated for the samples within and between groups, and a heatmap was drawn (**Figure 2B**). The R^2^ of each biological replicate was higher than 0.8, which met the requirements. The above-described data indicated that the transcriptome sequencing data were of high quality and could thus be used for the subsequent analysis.

Statistics on the number of sequences on the chromosomes can reflect the distribution of the sequenced lists on each chromosome from a macro perspective. Taking R1-1 as an example, the greatest number of reads appeared on chr13, followed by chr14, chr24 and chr17 (**Figure 2C**). The assembled transcriptome sequences were compared with six databases (NR, Swiss-Prot, Pfam, COG, GO and KEGG databases). A total of 43348 genes were ultimately annotated, and these accounted for 90.86% of the total number of unigenes (**Supplementary Table S3**). Among these, the highest number of unigenes was annotated in the NR database (43,307 unigenes, corresponding to 90.78% of the total), followed by the COG database (30,803, 64.57%) and GO database (28,842, 60.46%).

#### 3.1.2 DEGs analysis

As shown in **Table 1**, 4370, 3443 and 4069 DEGs were identified in Chinese red chestnut compared with ‘Songjiazao’ at the 3 developmental stages; among these, 2213, 2007 and 2007 were upregulated genes, and 2157, 1436 and 2062 were downregulated genes, respectively. R3 and R1 had the highest number of DEGs during the development of red chestnut: 7243 DEGs (3746 upregulated and 3497 downregulated DEGs). The greatest difference was detected between S3 and S2 at the early development period, with 6642 DEGs (2961 were upregulated and 3681 downregulated DEGs). A Venn diagram (**Supplementary Figure S1**) can intuitively show the overlap of DEGs identified from each comparison.

**Table 1:** Number of DEGs.

Comparison	Total DEGs	Upregulated	Downregulated
R2_*vs*_R1	4546	2724	1822
R3_*vs*_R1	7243	3746	3497
R3_*vs*_R2	5252	2156	3096
S2_*vs*_S1	6117	3334	2783
S3_*vs*_S1	6486	3223	3263
S3_*vs*_S2	6642	2961	3681
R1_*vs*_S1	4370	2213	2157
R2_*vs*_S2	3443	2007	1436
R3_*vs*_S3	4069	2007	2062

#### 3.1.3 GO classification and enrichment analysis of DEGs

**Figure 3:**
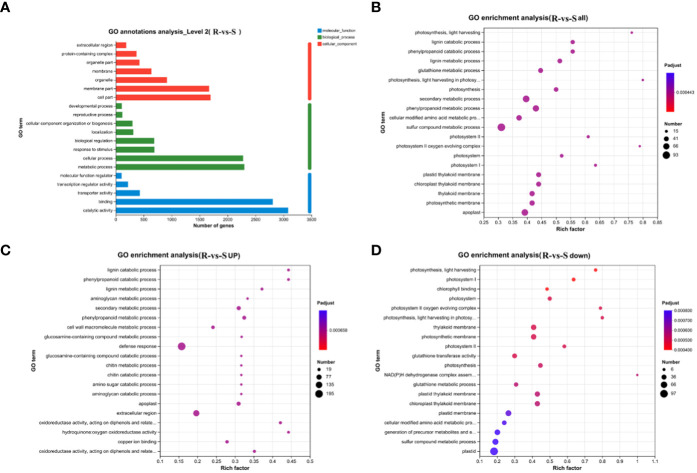
GO annotation and enrichment analysis. **(A)** GO annotation analysis. **(B)** GO enrichment analysis of DEGs; **(C)** GO enrichment analysis of Upregulated DEGs; **(D)** GO enrichment analysis of downregulated DEGs.

The annotation of the DEGs to the GO database successfully annotated 7801 DEGs identified from the comparison of the two varieties at 3 developmental stages to 3 biological processes. Among the 48 terms identified, 22 were associated with biological processes, 14 were annotated to molecular functions and 12 were associated with cellular component (**Supplementary Table S4**). **Figure 3A** shows the top 20 GO terms, and cell part, membrane part and organelle within the cellular component category, metabolic process and cellular process within the biological processes category, and catalytic activity and binding function within the molecular function category were annotated to high number of unigenes.

To analyze the specific functions of selected DEGs, a GO enrichment analysis of DEGs was further performed. The DEGs were mainly enriched in the sulfur compound metabolic process, secondary metabolic process, phenylpropanoid metabolic process, apoplast, thylakoid membrane, photosynthetic membrane, chloroplast thylakoid membrane, plastid thylakoid membrane and other pathways (**Figure 3B**). Among the DEGs, the downregulated genes (4055) were mainly enriched in the photosynthetic membrane, thylakoid membrane, photosynthesis, chloroplast thylakoid membrane, photosynthesis, light harvesting, photosystem I, chlorophyll binding, plastid and other pathways related to photosynthesis (**Figure 3C**). In contrast, the upregulated genes (4178) were mainly associated with the defense response, extracellular region, secondary metabolic process, phenylpropanoid metabolic process, lignin metabolic process, extracellular region, secondary metabolic process, phenylpropanoid metabolic process, lignin metabolic process, oxidoreductase activity and other pathways related to pigments, lignin metabolism, and antioxidants (**Figure 3D**). The results showed that photosynthesis was more highly enriched in green spines to provide nutrition for fruit development, whereas the red spines were more highly enriched in defense and secondary metabolic regulation.

#### 3.1.4 KEGG functional annotation and metabolic pathway enrichment analysis of DEGs

**Figure 4:**
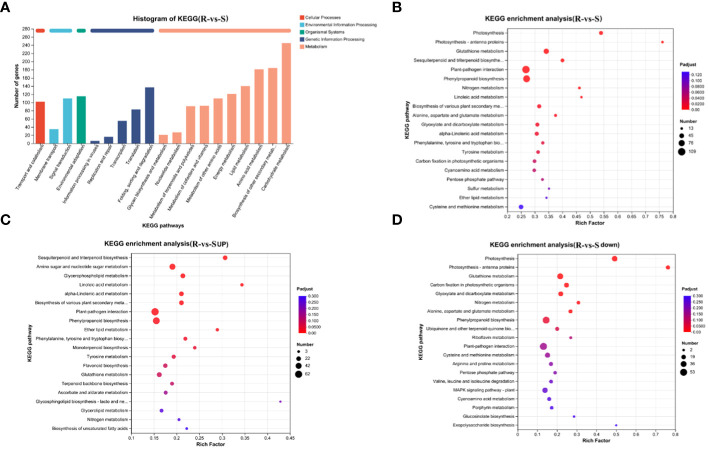
KEGG annotation and enrichment analysis of DEGs. **(A)** KEGG annotation analysis of DEGs; **(B)** Bubble map of the KEGG pathways enriched in DEGs; **(C)** Bubble map of KEGG pathways enriched in upregulated DEGs; **(D)** Bubble map of KEGG pathways enriched in downregulated DEGs.

KEGG pathway classification (**Figure 4A**) revealed that the annotated DEGs were distributed in 130 metabolic pathways, belonging to 5 categories. Among these, the metabolism category contained the largest number of genes and the largest number of secondary pathways (98). Further analysis of secondary pathways under the metabolism category was conducted. This category mainly included carbohydrate metabolism, biosynthesis of other secondary metabolites, amino acid metabolism and lipid metabolism, energy metabolism, metabolism of other amino acids, metabolism of cofactors and vitamins, metabolism of terpenoids and polyketides, nucleotide metabolism, glycan biosynthesis and metabolism and other secondary pathways. Flavonoids belong to a branch of secondary metabolism. We further analyzed the tertiary pathway involved in the biosynthesis of other secondary metabolites and found that the DEGs were annotated to 14 tertiary pathways, which included phenylpropanoid biosynthesis (map00940, 94), flavonoid biosynthesis (map00941, 31), anthocyanin biosynthesis (map00942, 2), isoflavonoid biosynthesis (map00943, 6), flavone and flavonol biosynthesis (map00944, 6), stilbenoid, diarylheptanoid and gingerol biosynthesis(map00945, 16), betalain biosynthesis(map00965, 1), isoquinoline alkaloid biosynthesis (map00950, 15), and other color-related pathways.

The processing of genetic information mainly included folding, sorting and degradation, translation, transcription, etc. The organismal systems mainly included environmental adaptation, the processing of environmental information mainly included signal transduction, and the membrane transport and cellular processes mainly included transport and catabolism.

Further KEGG enrichment analysis of the DEGs revealed that these genes that differential genes were mainly enriched in plant-pathogen interactions, phenylpropanoid biosynthesis, glutathione metabolism, glutathione metabolism, sesquiterpenoid and triterpenoid biosynthesis, photosynthesis, biosynthesis of various plant secondary metabolism, glyoxylate and dicarboxylate metabolism, alpha-linolenic acid metabolism and other pathways (**Figure 4B**). The bubble map of the KEGG pathways enriched in downregulated DEGs enrichment downregulation shows the top 20 pathways: photosynthesis, glutathione metabolism, carbon fixation in photosynthetic organisms, nitrogen metabolism, glyoxylate and dicarboxylate metabolism, phenylpropanoid biosynthesis and other pathways related to photosynthesis, carbon and nitrogen metabolism and flavonoid synthesis (**Figure 4C**). The pathways enriched in upregulated DEGs were mainly plant-pathogen interaction, phenylpropanoid biosynthesis, flavonoid biosynthesis, amino sugar and nucleotide sugar metabolism, sesquiterpenoid and triterpenoid biosynthesis, amino sugar and nucleotide sugar metabolism, sesquiterpenoid and triterpenoid biosynthesis, linoleic acid metabolism, alpha-linolenic acid metabolism and other pathways related to defense and the synthesis and metabolism of flavonoids, amino acids and other substances (**Figure 4D**). The results were similar to those obtained from the GO enrichment analysis, indicating that photosynthesis is enriched in green spines and that defense and metabolism regulation is enriched in red spines.

#### 3.1.5 Statistical analysis of transcription factors

**Figure 5:**
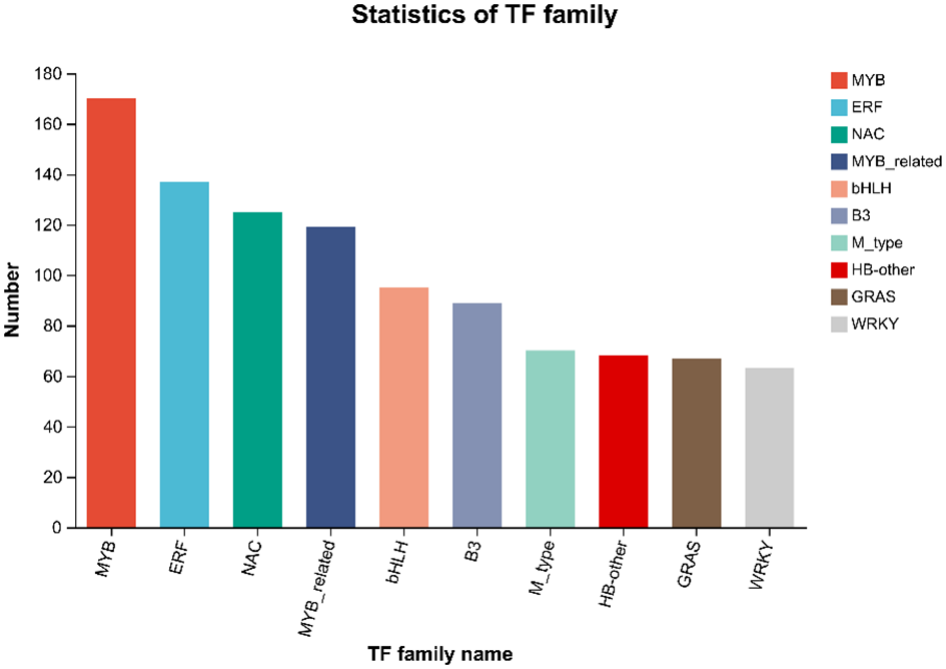
Statistics and analysis of transcription factor families.

Transcription factors can regulate plant development, secondary metabolism and stress resistance by binding to cis-acting elements in target gene promoters. By comparing unigenes to PlantTFDB, the unigenes were classified to TF families. The results of the TF prediction showed (**Figure 5**) that a total of 2758 genes were predicted, to belong to 48 transcription factor families. The top 5 transcription factor families with the greatest number of genes were *MYB* (170), *ERF* (137), *NAC* (125), *MYB*_related (119), and *bHLH* (95). In addition, 716 differentially expressed transcription factor-encoding genes were obtained, and those included 438 upregulated and 309 downregulated genes. *MYB* (31), *ERF* (29), *bHLH* (14), *NAC* (13) and *WRKY* (13) were the greatest upregulation, and *NAC* (31), *ERF* (30), *MYB* (27), *MYB*_related (14) and *WRKY* (10) were the highly downregulated transcription factors.

### 3.2 qRT-PCR validation

**Figure 6:**
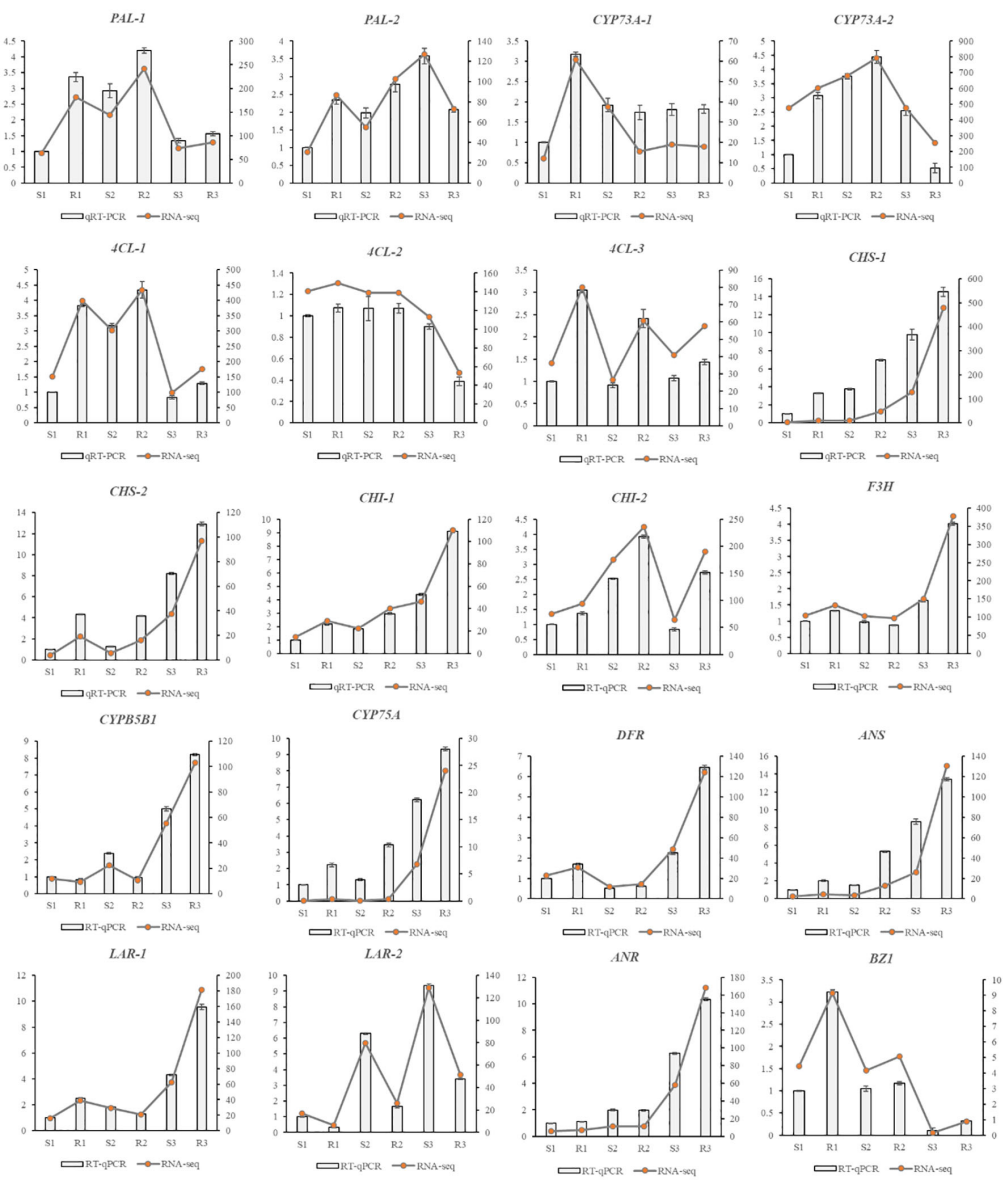
qRT-PCR results of DEGs associated with flavonoid synthesis.

The 20 genes selected for further validation were all DEGs with high expression levels and fold change in expression greater than 2, and related to flavonoid metabolism. The qRT-PCR results showed that the expression trends found for all selected genes were consistent with those identified from the transcriptome data (**Figure 6**). *PAL-1, 4CL-1, 4CL-3, CHS-1, CHS-2, CHI-1, CHI-2, CYP75A, DFR, ANS*, and *BZ1* were upregulated in all three periods, whereas *4CL-2* and *LAR-2* were downregulated. The other 7 genes were both upregulated and downregulated. *PAL-2* and *CYP73A-2* were upregulated in the first two periods, and downregulated in the third period, whereas the opposite pattern was observed for *CYP73A-1*. In addition, *F3H, CYP75B1, LAR-1* and *ANR* were not significantly upregulated in the first two periods but were significantly upregulated in the third period.

### 3.3 Analysis of differentially abundant metabolites

#### 3.3.1 Statistical analysis of metabolite differences

A total of 44 different metabolites were detected in this study, and these included 34 anthocyanins, 3 proanthocyanidins and 7 flavonoids (**Supplementary Table S5**). The 34 anthocyanins included 6 major anthocyanins, and the levels of cyanidin (10 types) (values of 276.20, 223.96, 182.40, 1.46, 0.01, and 0.86 ug/g were obtained in R1, R2, R3, S1, S2 and S3, were respectively), peonidin (types 5) (11.26, 4.84, 2.79, 0.04, 0.01, 0.02 ug/g) and pelargonidin (4 types) (2.62, 1.16, 0.48, 0.03, 0.02 and 0.02 ug/g) were relatively high. In contrast, the levels of delphinidin (4 types) (0.02, 0.04, 0, 0.02, 0.07 and 0.05 ug/g), petunidin (6 types) (0.10, 0.03, 0.07, 0.03, 0 and 0.01 ug/g) and malvidin (5 types) (0.06, 0.07, 0.01, 0.02, 0.01 and 0.01 ug/g) were low. To compare the metabolic components involved in pigment accumulation in the two different chestnut varieties, PCA was performed using the data set collected by UPLC-MS. The PC1×PC2 score plots revealed that the two different chestnut varieties could be clearly distinguished (**Supplementary Figure S2**). The PC1 and PC2 explained 38.3 and 35.9% of the variance, respectively, and clearly separated Chinese red chestnut from ‘Songjiazao’.

#### 3.3.2 Analysis of differences in anthocyanin accumulation

Moreover, during the development of Chinese red chestnut, the total anthocyanin content decreased (the levels in R1, R2 and R3 were 290.26 ug/g, 230.01 ug/g and 185.75 ug/g, respectively). The number of downregulated substances was markedly higher than the number of upregulated substances, and the downregulated substances were mainly malvidin, cyanidin, peonidin and petunidin, which also explained the gradual deepening of the red chestnut color and the gradual appearance of a brick red color. Two stages of early development were observed in ‘Songjiazao’, and S1-S2 contained a higher number of downregulated substances, which mainly included cyanidin, pelargonidin, and peonidin, and flavonoids, such as procyanidin, naringenin, kaempferol-3-O-rutinoside, afzelin, rutin, and quercetin-3-O-glucoside (isoquercetin). The total anthocyanin content decreased from 1.63 ug/g to 0.12 ug/g. In contrast, S2-S3 had a higher number of upregulated substances, which mainly included pelargonidin-3-O-glucoside and flavonoids such as naringin, kaempferol-3-o-rutin, afzelin, rutin, and quercetin-3-o-glucoside (isoquercetin). The total anthocyanin content increased to 0.96 ug/g. Compared with those in R and S, the levels of the upregulated substances increased significantly, the levels of peonidin, cyanidin, pelargonidin and malvidin increased significantly, and the levels of malvidin and delphinidin decreased (**Table 2**, **Supplementary Table S5**).

**Table 2:** Number of differentially abundant metabolites.

Comparison	Total metabolites	Upregulated	Downregulated
R2_*vs*_R1	18	5	13
R3_*vs*_R1	20	1	19
R3_*vs*_R2	18	3	15
S2_*vs*_S1	22	4	18
S3_*vs*_S1	17	4	13
S3_*vs*_S2	15	11	4
R1_*vs*_S1	27	23	4
R2_*vs*_S2	29	25	4
R3_*vs*_S3	27	20	7

#### 3.3.3 KEGG enrichment analysis of differentially abundant metabolites

**Figure 7:**
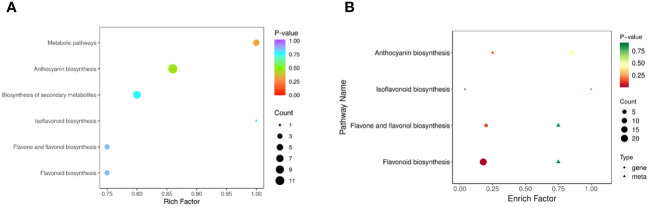
KEGG enrichment analysis. **(A)** KEGG pathways enriched in differentially abundant metabolites. **(B)** Combined KEGG enrichment analysis.

A KEGG enrichment analysis (**Figure 7A**) of differentially abundant metabolites was performed, and the results revealed that these metabolites were mainly enriched in 6 pathways. Among these pathways, the anthocyanin pathway was the most abundant, followed by biosynthesis of secondary metabolites, metabolic pathways, flavone and flavonol biosynthesis, flavonoid biosynthesis and isoflavonoid biosynthesis.

### 3.4 Metabolic pathway analysis

#### 3.4.1 Anthocyanin synthesis pathway

The results from the combined analysis showed that 4 KEGG pathways were shared by the transcriptome and metabolome (**Figure 7B**). Flavonoid biosynthesis, anthocyanin biosynthesis, flavone and flavonol biosynthesis, and isoflavonoid biosynthesis are pathways involved in flavonoid synthesis.

**Figure 8:**
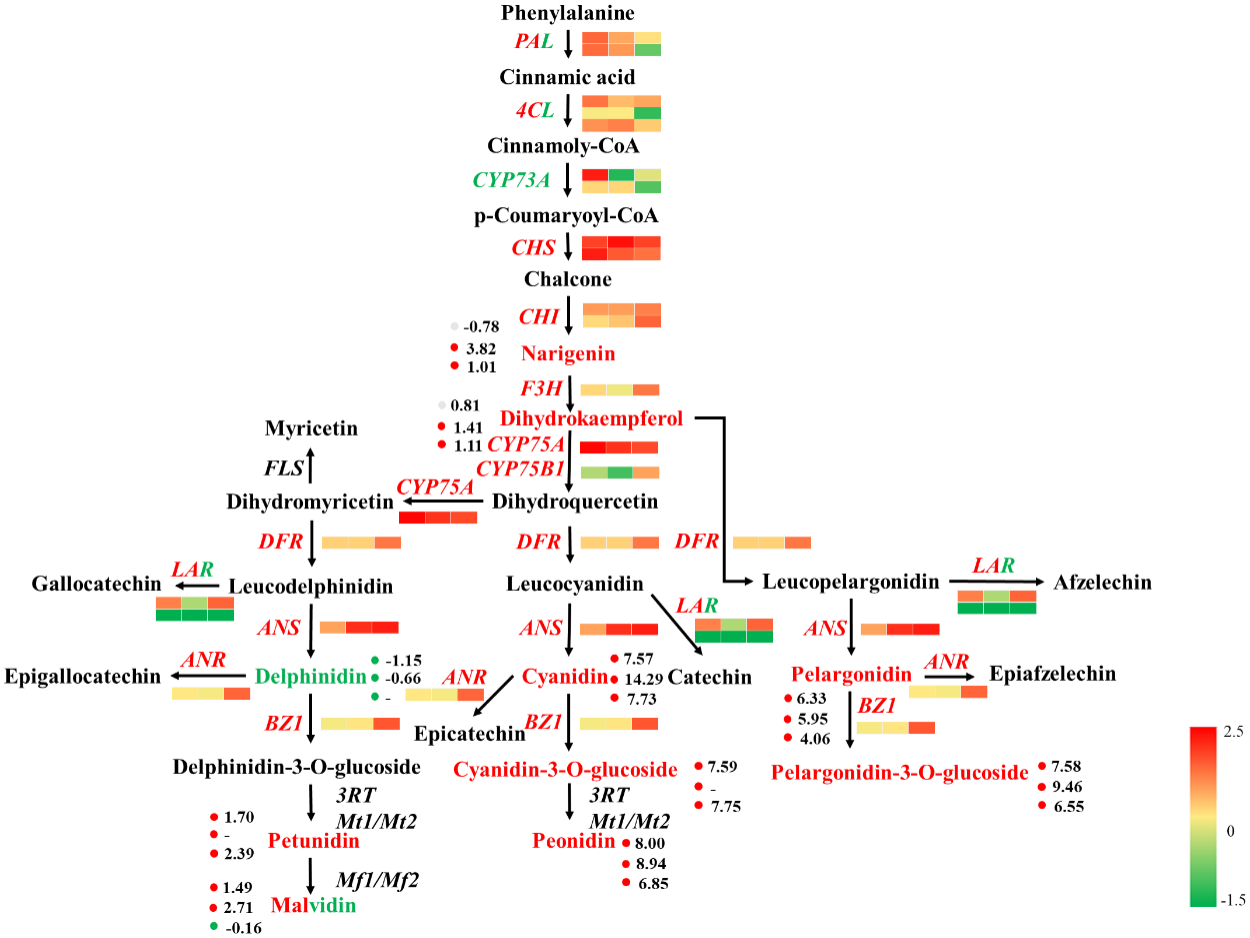
Anthocyanin synthesis pathway. Red indicates upregulated genes or metabolites, green indicates downregulated genes or metabolites, and red plus green indicates both upregulated and downregulated genes or metabolites. The gene heatmap from left to right show the results from the R1-*vs*.-S1, R2-*vs*.-S2, and R3-*vs*.-S3 comparisons.

According to the results of the combined analysis, the anthocyanin synthesis pathway was selected for further analysis. According to the annotation of the DEGs and differentially abundant metabolites identified form the R-*vs*.-S comparison to the anthocyanin biosynthesis pathway in the KEGG database (using log_2_FC as the parameter), a map of the DEGs and differentially accumulated metabolites in the anthocyanin synthesis pathway of Chinese red chestnut was drawn (**Figure 8**). Six types of anthocyanins were found in the spines of Chinese chestnut, and 3 anthocyanin synthesis pathways that compete with each other also occur in these spines. Compared with those in ‘Songjiazao’, the levels of 6 pigments were increased in Chinese red chestnut spines. Among those pigments, the levels of cyanidin, peonidin, pelargonidin and malvidin were significantly increased, indicating that the cyanidin and pelargonidin pathways were enhanced; in contrast, the delphinidin pathway was weaker, and a greater amount of delphinidin pigments was transformed into malvidin pigments. In addition, the levels of flavonoids related to anthocyanin synthesis, such as naringenin and dihydrokaempferol, were also significantly increased. During the synthesis of flower pigments, the expression of the *CHS, CHI, F3H, CYP75A, CYP75B1, DFR*, and *ANS* genes clearly increased, that of *CYP73A* clearly decreased, and that of *PAL, 4 CL* and *LAR* both increased and decreased. The synthesized anthocyanin can be converted into anthocyanidin or epicatechin. In Chinese red chestnut, the upregulation of the *BZ1* gene increases the corresponding anthocyanidin content, and the upregulation of the *ANR* gene also promotes the conversion of anthocyanin to epicatechin. In particular, the *ANR* increased significantly in the third period.

#### 3.4.2 Photosynthetic pathways

**Figure 9:**
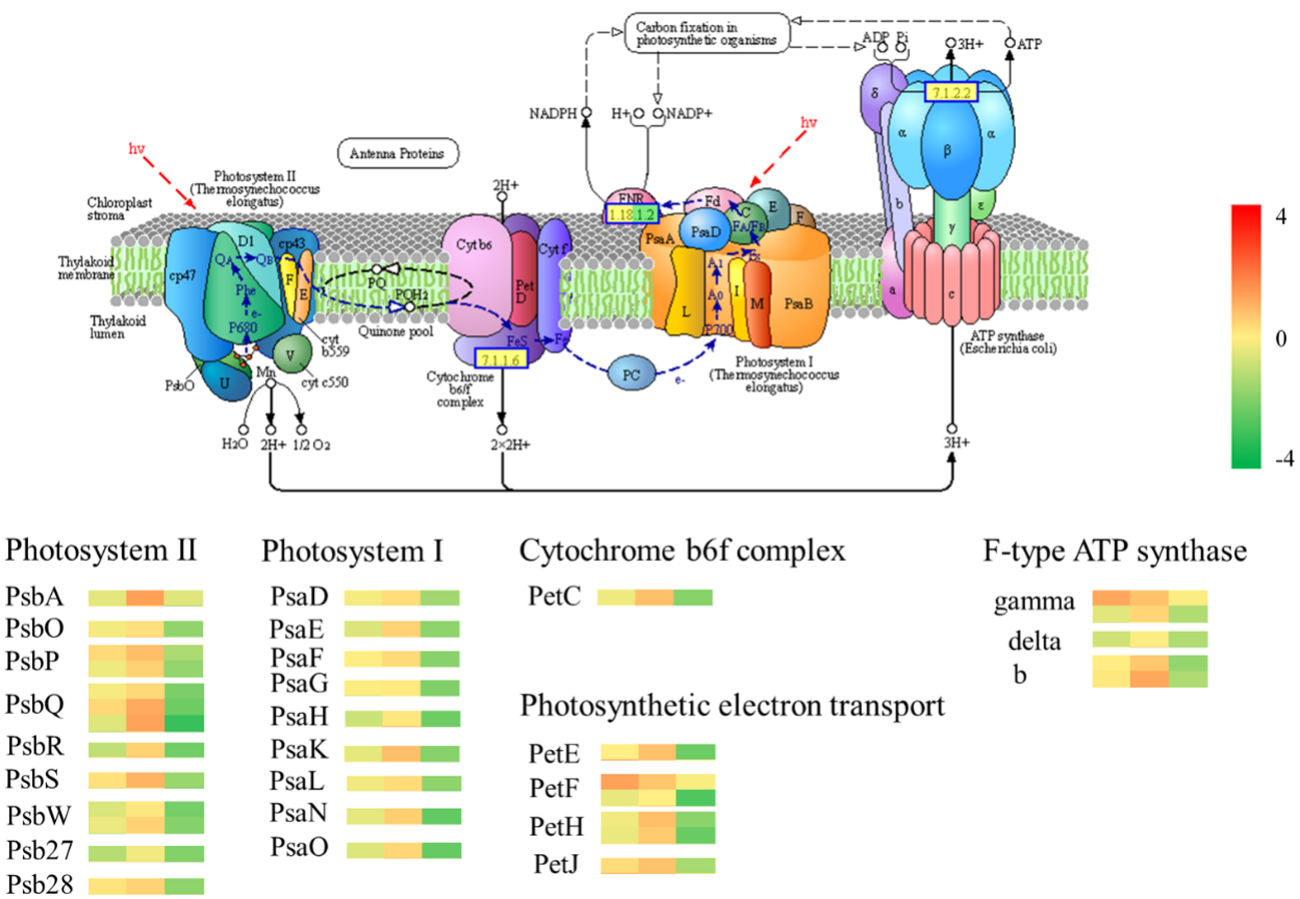
Photosynthesis pathway. The gene heatmap from left to right show the results from the R1-*vs*.-S1, R2-*vs*.-S2, R3-*vs*.-S3 comparisons.

The GO analysis and KEGG analysis of transcriptomic data revealed significant differences in photosynthesis pathways between Chinese red chestnut and ‘Songjiazao’; thus, the photosynthesis pathways were selected for further analysis. Five multicomponent complexes photosystem II (PS II), the cyto-chrome b6f complex, photosystem I (PS I), photosynthetic electron transport and F-type ATP synthase, work together to accomplish light-dependent energy-producing PET reactions (**Figure 9**). The experimental results showed that the photosynthetic performance of red chestnut spines gradually decreased during development compared with that of green spines, and this difference was particularly obvious in the third stage. All 34 DEGs in the photosynthesis pathway (map00195) were downregulated. Thirteen downregulated DEGs (*PsbO, PsbP, PsbQ, PsbR, PsbS,PsbW, Psb27* and *Psb28*) were annotated to PS II, and 9 downregulated DEGs (*PsaD, PsaE, PsaF, PsaG, PsaH, PsaK, PsaL, PsaN* and *PsaO*) were associated with PI. The *PetC* gene in the cyto-chrome b6f complex was downregulated, and the expression of the *PetE, PetF, PetH* and *PetJ* genes involved in photosynthetic electron transfer in the KEGG photosynthesis pathway was downregulated (6), resulting in decreases in the levels of PC, Fd, FNR, and cytc6 proteins. Moreover, these alterations lead to NADPH and ATP deficiency and decreased photoassimilate accumulation in red chestnut (Berry et al., 2013). The gamma, delta, and b genes of F-type ATPases were also downregulated (5). Moreover, 24 of the 29 DEGs in the carbon fixation in photosynthetic organisms (map00710) pathway were downregulated and 5 were upregulated. All 16 DEGs encoding photosynthesis antenna proteins were downregulated. Starch and sucrose metabolism (map00500) was enriched in 30 downregulated DEGs of the 53 DEGs. The downregulation of these genes may lead to a decrease in the levels of D-glucose-6p (glucose), D-glucose, a-D-glucose-1P cellobiose, sucrose and trehalose-6p trehalose. Compared with those of green spines, the photosynthetic characteristics of red spines were significantly weakened, and the levels of substances related to carbon fixation and synthetic photosynthesis were reduced.

#### 3.4.3 Plant-pathogen interaction pathway

**Figure 10:**
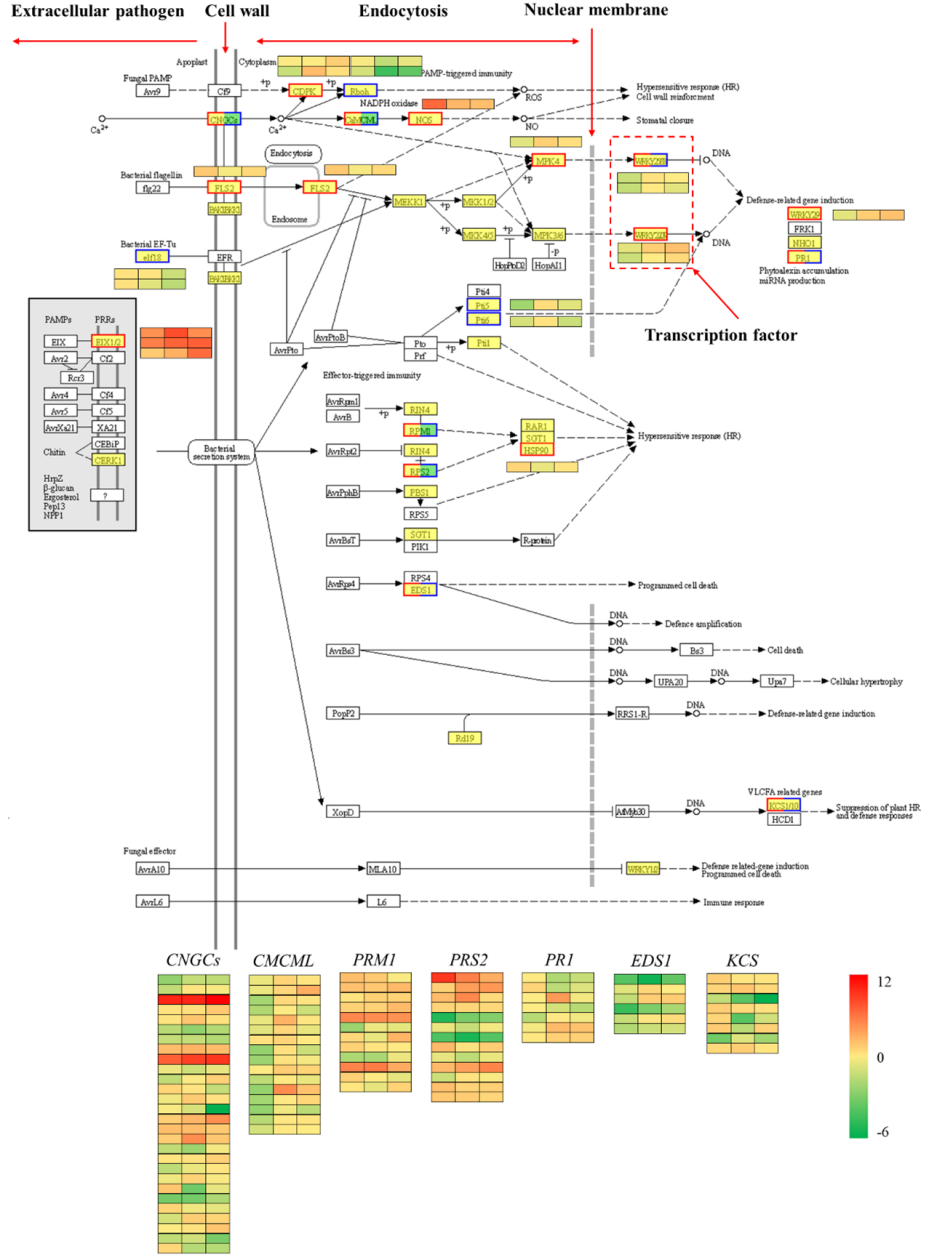
Plant-pathogen interaction pathway. Red indicates upregulation, blue indicates downregulation, and both red and blue indicates both up- and downregulation.

The plant-pathogen interaction pathway was selected for further analysis because it was clearly enriched in the plant defense response according to the differential transcript expression analysis. Proteins produced by pathogenic agents outside the cell can be recognized by the plant cell wall or transmembrane protein receptors within the membrane. For example, the *FLS2* gene can recognize bacterial flagellin (*flg22*), and the bacterial EF-TU gene (*elf18*) is recognized by the plant EF receptor (EFR). These protein receptors recognize the signals of pathogenic microorganisms, transport them into the cell and then activate a series of other proteins, such as protein kinases, to transmit the related signals along. These signals ultimately stimulate plant cells to initiate related hypersensitivity responses, such as increased secretion of reactive oxygen species and increased expression of defense-related genes (Chen et al., 2023). The comparison of red chestnut with ‘Songjiazao’, identified 109 DEGs in the plant-pathogen interaction pathway. As shown in **Figure 10**, the expression of defense-related genes, such as *CNGCs*, *CDPK*, *CaMCML*, *NOS*, *FLS2*, and *MPK4*, in the spines of red chestnut was significantly upregulated, and these genes subsequently activated the *WRKY33/WRKY29/WRKY22* TFs, and thereby the expression of downstream immune defense-related genes.

Based on the above analysis, we concluded that the difference in expression between red chestnut and green chestnut may be related to a photoprotective mechanism (Velitchkova et al., 2020; Lou et al., 2022). Due to the differential expression of structural genes or transcription factors, spines appear red and exhibit reduced photosynthesis, thus, excess light will cause photodamage to plant cells, which indicates that the photoprotection mode is turned on. In particular, at the early growth stage, DEGs related to defense-related pathways were clearly enriched in the red chestnut cultivar. At the late stage of growth and development, the protective mechanism weakens with decreases in the anthocyanin content.

## 4 Discussion

At present, the combination of metabolome and transcriptome analyses is an effective means for identifying metabolite-related functional genes, and is widely used in studies of plant growth and development, physiological regulation, and stress responses, among other processes. Transcriptome sequencing can identify many DEGs and regulatory metabolic pathways, and metabolites can reflect biochemical reactions in plants relatively effectively. The results reveal a bridge between the genotype and the phenotype (Zhang et al., 2020a). Zou et al. (2020) reported that differences in the taste of different loquat varieties may be affected by differences in the expression of genes and the levels of carbohydrates, organic acids, amino acids and phenols. Cheng et al. (2014) and Fu et al. (2021) reported that cyanidins are the main anthocyanins responsible for the pink and red petals of Camellia japonica and *Prunus persica*. Zhang et al. (2019) revealed the metabolic and molecular mechanism by which intense light regulates the uneven accumulation of anthocyanins in tomato fruits. Cho et al. (2016) analyzed the regulation of potato pigments. Dong et al. (2019) investigated the molecular mechanism of the formation of anthocyanins in asparagus, and Wang et al. (2017) studied the components and formation of anthocyanins in *Ficus carica*. In this study, the spines of Chinese red chestnut with red spines and those of ‘Songjiazao’ chestnut with green spines were analyzed via transcriptome sequencing and anthocyanin metabolomics, and the results were analyzed together. A total of 109.26 Gb of clean base data were obtained by sequencing, and the data from each sample was at least 6.07 G. GO and KEGG analyses revealed that photosynthesis was more highly enriched in green spines compared with red spines. Compared with those of green spines, the photosynthetic characteristics of red spines were significantly weakened. Most of the DEGs related to photosynthesis, carbon fixation in photosynthetic organisms, photosynthesis antenna proteins, and starch and sucrose metabolism pathways were downregulated. Defense and metabolic regulation-related pathways, such as plant-pathogen interactions, phenylpropanoid biosynthesis, flavonoid biosynthesis, amino sugar and nucleotide sugar metabolism, sesquiterpenoid and triterpenoid biosynthesis, linoleic acid metabolism, alpha-linolenic acid metabolism and other pathways, were more strongly enriched in red spines. The DEGs in these pathways were more highly upregulated. Through annotation of the differential gene to the KEGG database, the sequences of *PAL, CYP73A, 4CL, CHS, CHI, F3H, CYP75A, CYP75B1, DFR, LAR, ANS, ANR, BZ1* and other genes related to anthocyanin synthesis were screened. Among those genes, *ANS* and *BZ1* were found to be directly related to leucoanthocyanidin and anthocyanins. Qi et al. (2014) showed that introduction of the exogenous mulberry *ANS* gene can make Arabidopsis exhibit a light red color, and its overexpression can significantly increase the amount of anthocyanin. Du et al. (2017) and Ford et al. (1998) showed that *BZ1* encodes UDP glucosyltransferase (UFGT), which can glucosylate anthocyanins in fruits during ripening and convert these metabolites into stable anthocyanidins. In this study, *ANS* and *BZ1* were significantly greater in red spines than in green spines. A total of 44 different metabolites involved in anthocyanin metabolism were detected, and these included 34 anthocyanins, 3 proanthocyanidins and 7 flavonoids. The 34 anthocyanins were divided into 6 major anthocyanins. Compared with those in ‘Songjiazao’, the levels of peonidin, cyanidin, pelargonidin and malvidin were significantly increase in red chestnut, whereas the levels of malvidin and delphinidin were decreased. Through comparative omics analysis, the correlations between the variations in the anthocyanin contents and the specific expression of one or more genes was explored, and the correlation between the color of different spines and the types and levels of anthocyanins were analyzed, which provided the basis for improving the development and cultivation of chestnuts with high ornamental value and edible value.

Flavonoids are important products of plant secondary metabolism and play an important role in plant color. Flavonoids, especially anthocyanins, can cause plants to exhibit various changes such as yellow, red and blue coloration (Mazza and Miniati, 1993; Wang et al., 2017; Dong et al., 2019). Leaves that contain higher proportions of chlorophyll, anthocyanins and carotenoids appear green, red, orange, blue or yellow. This study revealed that there were 34 types of anthocyanins in the spines of Chinese red chestnut, and the levels of cyanidin, peonidin and pelargonidin were greater, whereas the levels of delphinidin, petunidin and malvidin were lower. And the types and the levels of cyanidin were the highest, which also explained the red color of the spines of Chinese red chestnut. This finding is consistent with the results form a study of *Ficus carica* conducted by Wang et al. (2017). Morever, during the development of red chestnut, the levels of some delphinium pigments, petunia pigments and mallow pigments increased, whereas those of cyanidin, paeoniflorin and pelargonidin decreased. Previous studies have shown that the three pathways involved in anthocyanin synthesis exhibit a competitive relationship (Ververidis et al., 2007). Therefore, during the development process of Chinese red chestnut, the cyanidin and pelargonidin pigment pathway was downregulated, the delphinidin pigment pathways were slightly upregulated, and the total pigment content was reduced, which resulted in the red spines becoming darker in color during development and presenting a brick red color at the later stage.

In addition, small amounts of delphinidin-3-O-rutinoside, delphinidin-3-o-rutinoside-5-o-glucoside, delphinidin-3-O-sambubioside, and delphinidin-3-O-arabinoside were detected in ‘Songjiazao’, and small amounts of delphinidin-3-O-rutinoside and delphinidin-3-O-arabinoside were detected in Chinese red chestnuts. This finding indicates that chestnut spiny buds can indeed synthesize blue anthocyanins. The failure of spines to show a blue color may be due to the low content of delphinidin, or be affected by other pigment colors with higher content, or the lack of expression of related genes or transcription factor, such as *MYB*, *bHLH*, *WRKY* and others. *MYB* and *bHLH* transcription factors and WD40 proteins usually form a highly conserved MYB-bHLH-WD40 (MBW) transcription complex that regulates the biosynthesis of anthocyanins in plants (Chen et al., 2019b; Li et al., 2020; Yang et al., 2020). In this study, 170 *MYB*, 95 *bHLH*, and 63 *WRKY* transcription factors were also identified, and these included 51 *MYB*, 21 *bHLH*, and 23 *WRKY* differentially expressed transcription factor, which may be closely related to color change. However, this hypothesis needs further research. Therefore, in future investigations and research, attention should be given to determining whether materials that exhibit in the natural variation can be used to breed new varieties of blue and purple spines. Chestnuts with blue and purple spines could be cultivated by selective breeding or molecular breeding, and a new type of chestnut with great ornamental value and value for use in food should be cultivated.

In addition, transcription factors that play important roles in regulating plant growth and development and biological and abiotic stress responses, such as *ERF, MYB, NAC, MYB_related*, and *WRKY*, were screened via transcription factor analysis. GO and KEGG enrichment analyses revealed that the red spines of Chinese red chestnut were more strongly involved in defense and secondary metabolic regulation. Among the upregulated pathways, the plant-pathogen interaction pathway (**Figure 10**) contained 109 DEGs. In this study, two *WRKY33* transcription factors (one upregulated and one downregulated), one *WRKY29* (upregulated) and one *WRKY22* transcription factor (upregulated) were screened. Studies have shown that when induced by external stimuli in plants, *WRKY* transcription factors are regulated by a cascade of defense signaling networks that bind with the promoters of downstream genes to regulate their expression and enhance plant defense (Rushton et al., 2010; Song et al., 2023). The effects of these three *WRKY* genes on drought and cold resistance in other plants have been reported (Guo et al., 2022; Li et al., 2022; Zhao et al., 2022; Song et al., 2023). Therefore, we speculate that these four *WRKY* transcription factors play important regulatory roles in the resistance of chestnut to stress, which is the direction of our next study.

## 5 Conclusion

(1) A total of 119.34 G of clean base data were obtained in this study, and at least 6.07 G was obtained from each sample. GO and KEGG analyses revealed that photosynthesis was more highly enriched in green spines than in red spines. Compared with those of green spines, the photosynthetic characteristics of red spines were significantly weakened. Most of the DEGs related to photosynthesis, carbon fixation in photosynthetic organisms, photosynthesis antenna proteins, and starch and sucrose metabolism were downregulated in red spines compared with green spines, and defense and metabolism regulation was more highly enriched in red spines compared with green spines. Most of the DEGs related to phenylpropanoid biosynthesis, flavonoid biosynthesis, amino sugar and nucleotide sugar metabolism, sesquiterpenoid and triterpenoid biosynthesis, linoleic acid metabolism, alpha-linolenic acid metabolism and other pathways were upregulated. The analysis showed that the change in spine color promoted the red chestnut to turn on the photoprotection mode, especially at the early growth stage, and the DEGs were clearly enriched in defense-related pathways. The sequences of anthocyanin structural synthesis-related genes such as *PAL*, *CYP73A*, *4CL*, *CHS*, *CHI*, *F3H*, *CYP75A*, *CYP75B1*, *DFR*, *LAR*, *ANS*, *ANR*, and *BZ1* and 4 *WRKY* transcription factors associated with stress resistance were obtained by annotating the DEGs to the KEGG database. The qRT-PCR results showed that the expression trend of 20 candidate anthocyanin synthesis-related genes was the same as that found from the transcriptome results, indicating that the transcriptome results were highly reliable.
(2) A total of 44 different metabolites involved in anthocyanin metabolism, including 34 anthocyanins, 3 proanthocyanidins and 7 flavonoids, were detected. The 34 anthocyanins were divided into 6 major anthocyanins, and relatively high levels of cyanidin, peonidin and pelargonidin and relatively low levels of delphinidin, petunidin and malvidin were found in red spines, which also explains the red color of Chinese red chestnut spines. In addition, during the maturation of red chestnut, the total anthocyanin content decreased, and the contents of some delphinium pigments, petunia pigments and malva pigments increased, gradually increasing the depth of the color of the red spines. Compared with those in ‘Songjiazao’, the levels of cyanidin, peonidin, pelargonidin and malvidin in Chinese red chestnut spines were significantly increased, indicating that the cyanidin and pelargonidin pathways were enhanced, whereas the delphinidin pathways were weakened, and more delphinidin pigments were transformed into malvidin. During the synthesis of flower pigments, the expression of the *CHS*, *CHI*, *F3H*, *CYP75A*, *CYP75B1*, *DFR*, and *ANS* genes clearly increased, that of *CYP73A* clearly decrease, and that of *PAL, 4CL* and *LAR* both increased and decreased. The synthesized anthocyanin can be converted into anthocyanidin or epicatechin. In Chinese red chestnut, the upregulation of the *BZ1* gene increases the corresponding anthocyanidin content, and the upregulation of the *ANR* gene also promotes the conversion of anthocyanin to epicatechin.

## Data availability statement

The original contributions presented in the study are included in the article/**Supplementary Material**. Further inquiries can be directed to the corresponding authors.

## Author contributions

QQ: Writing – original draft, Data curation, Conceptualization. YG: Writing – original draft, Data curation, Conceptualization. QL: Writing – review & editing, Conceptualization.
