# Supplementary material for: Metabolic and molecular mechanisms of spine color formation in Chinese red chestnut
Source: Front Plant Sci. 2024 May 21;15:1377899. doi: 10.3389/fpls.2024.1377899 (PMC11148441; doi:10.3389/fpls.2024.1377899)
Supplement: Supplementary file 1 [file DataSheet_1.docx]

Table S1 Primer sequences for the target genes

| Gene | Description | Sequence  (5’->3’) | Length (bp) |
| --- | --- | --- | --- |
| *PAL-1* | Phenylalanine ammonia-lyase activity | F: CGAGTTCAGGAAGCCATTGG  R: CTTTACCAGACGCCATGCTC | 179 bp |
| *PAL-2* |  | F: CAGCTCTCCCTCTCCCAAAA | 154 bp |
|  |  | R: ACCCCTGAAAATTGCGACAC |  |
| *CYP73A-1* | Cytochrome P450 CYP73A100-like | F: AGTGGTAAATGCATGGTGGC | 161 bp |
|  |  | R: AGCTCCTCCTACCCATACCA |  |
| *CYP73A-2* |  | F: TCCTCTGCTTGTTCCACACA | 237 bp |
|  |  | R: ATTCCAGGGCAGCTTCTTCT |  |
| *4CL-1* | 4-Coumarate--CoA ligase-like 9 | F: CTGGGGAAGGAGCTTGAAGA | 208 bp |
|  |  | R: GCAAAGAGGCACCAGTTTCA |  |
| *4CL-2* |  | F: TCCCAAACCATCTCCCACTC | 160 bp |
|  |  | R: ATGCCCAACTTGTCCAAACC |  |
| *4CL-3* |  | F: CAAACCTGCTCTCATCGACG | 206 bp |
|  |  | R: GGTTGGTTGTTGAGGCGATT |  |
| *CHS-1* | Chalcone synthase 1 | F: GGGTGCTCGTGTACTTGTTG | 151 bp |
|  |  | R: AACCTCAGGAATCGGGTCAG |  |
| *CHS-2* |  | F: CTTCCGAATCACCAAGAGCG | 224 bp |
|  |  | R: TGATTGCCTTTGTTGCTGCT |  |
| *CHI-1* | Chalcone isomerase-like protein 2 | F: GATGACTTCTTCGAGGCCCT | 172 bp |
|  |  | R: CTTTCTCCAATGCTGCCTCC |  |
| *CHI-2* |  | F: ATCGTTACAGGCCCCTTTGA | 223 bp |
|  |  | R: CCTTGGGTGATTGCGTGAAA |  |
| *F3H* | Flavanone 3-hydroxy-lase | F: CAGGATCAGGTTGGTGGTCT | 171 bp |
|  |  | R: GCTGCTGTTTGAGTTCACCA |  |
| *CYP75B1* | Flavonoid 3'-monooxygenase | F: AATATGTTCACGGCAGGCAC | 188 bp |
|  |  | R: GTTTCCTTGACCACAGCCTG |  |
| *CYP75A* | Flavonoid 3',5'-hydroxylase 1 | F: TGGGCACTTACCGAGATGTT | 175 bp |
|  |  | R: AGTTTAGTGGTGTGGAGGGG |  |
| *DFR* | Dihydroflavonol 4-reductase | F: CCAAAGGCAGAGGGAAGGTA | 234 bp |
|  |  | R: CTTGGCTCGGCATGTTTCAA |  |
| *ANS* | Anthocyanidin synthase | F: TTCCTGGCCTGCAACTTTTC | 150 bp |
|  |  | R: TCTCCTTGTTCACCAGTCCC |  |
| *LAR-1* | Leucoanthocyanidin reductase | F: GCCCATCCCACATAGCATTG | 155 bp |
|  |  | R: AGTCCTTGAAGCACTCGTCA |  |
| *LAR-2* |  | F: TGTTTTACAAAGGCCGAGGT | 101 bp |
|  |  | R: AGGTAGACTTGAATACATGCACT |  |
| *ANR* | Anthocyanidin reductase | F: TGTGATCCCGACTCTCATGG | 168 bp |
|  |  | R: CGGCAAACATCCTCCACATG |  |
| *BZ1* | Anthocyanidin 3-O-glucosyltransferase UFGT | F: GTTGCCTTTCCCTTGCTCTC | 154 bp |
|  |  | R: CTCCGATAGCACCTCCTCAG |  |
| *Actin* | Internal reference gene | F: ATTCACGAGACCACCTACA | 174 bp |
|  |  | R: TGCCACAACCTTAATCTTCAT |  |

Table S2 Output data statistics of 18 samples

| Sample | Raw reads | Raw bases | Clean reads | Clean bases | Error rate (%) | Q20 (%) | Q30 (%) | GC content (%) |
| --- | --- | --- | --- | --- | --- | --- | --- | --- |
| R1_1 | 45821034 | 6918976134 | 44538430 | 6561816462 | 0.0268 | 97.36 | 92.6 | 44.94 |
| R1_2 | 48010950 | 7249653450 | 46468304 | 6818096589 | 0.0267 | 97.39 | 92.69 | 45.02 |
| R1_3 | 48526100 | 7327441100 | 46964016 | 6923709231 | 0.0268 | 97.37 | 92.6 | 44.85 |
| R2_1 | 48699190 | 7353577690 | 46839376 | 6812970489 | 0.0265 | 97.46 | 92.89 | 45.07 |
| R2_2 | 44725168 | 6753500368 | 43381236 | 6396292890 | 0.0271 | 97.21 | 92.3 | 44.57 |
| R2_3 | 45182418 | 6822545118 | 43565680 | 6420202660 | 0.027 | 97.24 | 92.38 | 44.59 |
| R3_1 | 43164024 | 6517767624 | 41564586 | 6098932209 | 0.0268 | 97.34 | 92.57 | 45 |
| R3_2 | 49250882 | 7436883182 | 47887224 | 7041840400 | 0.0263 | 97.54 | 93.03 | 44.25 |
| R3_3 | 45692002 | 6899492302 | 44153184 | 6470356940 | 0.0267 | 97.38 | 92.69 | 44.14 |
| S1_1 | 42891252 | 6476579052 | 41127350 | 6067273187 | 0.0266 | 97.43 | 92.77 | 44.7 |
| S1_2 | 49749040 | 7512105040 | 48162566 | 7077719380 | 0.0273 | 97.15 | 92.14 | 44.9 |
| S1_3 | 53064924 | 8012803524 | 51749978 | 7614657428 | 0.0265 | 97.47 | 92.87 | 45.01 |
| S2_1 | 45611422 | 6887324722 | 44046838 | 6462787954 | 0.0266 | 97.42 | 92.83 | 45.24 |
| S2_2 | 43035632 | 6498380432 | 41650630 | 6157559698 | 0.0267 | 97.35 | 92.63 | 44.72 |
| S2_3 | 49417388 | 7462025588 | 47825182 | 7024770899 | 0.0266 | 97.43 | 92.8 | 45.13 |
| S3_1 | 45177800 | 6821847800 | 43215464 | 6392259316 | 0.0267 | 97.36 | 92.62 | 44.46 |
| S3_2 | 45840236 | 6921875636 | 44078000 | 6536794689 | 0.0275 | 97.06 | 91.94 | 44.53 |
| S3_3 | 45033782 | 6800101082 | 43667266 | 6461460696 | 0.0265 | 97.47 | 92.86 | 44.63 |

Table S3 Statistics of the transcriptome annotation results

|  | Expre_Gene number (percent) | Expre_Transcript number (percent) | All_Gene number (percent) | All_Transcript number (percent) |
| --- | --- | --- | --- | --- |
| GO | 21981(0.7223) | 23827(0.7364) | 28842(0.6046) | 31550(0.6185) |
| KEGG | 11210(0.3684) | 12285(0.3797) | 13868(0.2907) | 15421(0.3023) |
| COG | 24569(0.8073) | 26734(0.8263) | 30803(0.6457) | 33850(0.6636) |
| NR | 29694(0.9757) | 31719(0.9804) | 43307(0.9078) | 46603(0.9137) |
| Swiss-Prot | 22791(0.7489) | 24870(0.7687) | 27909(0.585) | 30774(0.6033) |
| Pfam | 23447(0.7705) | 25564(0.7901) | 28742(0.6025) | 31577(0.6191) |
| Total_anno | 29706(0.9761) | 31729(0.9807) | 43348(0.9086) | 46644(0.9145) |
| Total | 30432(1.0) | 32354(1.0) | 47706(1) | 51007(1) |

Table S4 Results of GO database annotation

| Term category | GO ID | Description | Gene ratio |
| --- | --- | --- | --- |
| Biological process | GO:0001906 | cell killing | 5/7801 |
|  | GO:0002376 | immune system process | 19/7801 |
|  | GO:0015976 | carbon utilization | 5/7801 |
|  | GO:0065007 | biological regulation | 687/7801 |
|  | GO:0008152 | metabolic process | 2298/7801 |
|  | GO:0051704 | multi organism process | 103/7801 |
|  | GO:0040011 | locomotion | 4/7801 |
|  | GO:0022414 | reproductive process | 116/7801 |
|  | GO:0000003 | reproduction | 4/7801 |
|  | GO:0008283 | cell population proliferation | 5/7801 |
|  | GO:0071840 | cellular component organization or biogenesis | 299/7801 |
|  | GO:0009987 | cellular process | 2277/7801 |
|  | GO:0032502 | developmental process | 107/7801 |
|  | GO:0032501 | multicellular organismal process | 38/7801 |
|  | GO:0040007 | growth | 7/7801 |
|  | GO:0048511 | rhythmic process | 5/7801 |
|  | GO:0051179 | localization | 314/7801 |
|  | GO:0022610 | biological adhesion | 3/7801 |
|  | GO:0098754 | detoxification | 7/7801 |
|  | GO:0023052 | signaling | 1/7801 |
|  | GO:0019740 | nitrogen utilization | 1/7801 |
|  | GO:0050896 | response to stimulus | 692/7801 |
| Cellular component | GO:0031974 | membrane-enclosed lumen | 14/7801 |
|  | GO:0032991 | protein-containing complex | 370/7801 |
|  | GO:0044425 | membrane part | 1670/7801 |
|  | GO:0044421 | extracellular region part | 25/7801 |
|  | GO:0044422 | organelle part | 424/7801 |
|  | GO:0043226 | organelle | 917/7801 |
|  | GO:0016020 | membrane | 639/7801 |
|  | GO:0030054 | cell junction | 11/7801 |
|  | GO:0005576 | extracellular region | 187/7801 |
|  | GO:0009295 | nucleoid | 3/7801 |
|  | GO:0044464 | cell part | 1697/7801 |
|  | GO:0099080 | supramolecular complex | 32/7801 |
| Molecular function | GO:0045182 | translation regulator activity | 29/7801 |
|  | GO:0140110 | transcription regulator activity | 218/7801 |
|  | GO:0005198 | structural molecule activity | 90/7801 |
|  | GO:0044183 | protein folding chaperone | 11/7801 |
|  | GO:0140104 | molecular carrier activity | 4/7801 |
|  | GO:0016209 | antioxidant activity | 77/7801 |
|  | GO:0005215 | transporter activity | 430/7801 |
|  | GO:0098772 | molecular function regulator | 104/7801 |
|  | GO:0140299 | small molecule sensor activity | 3/7801 |
|  | GO:0005488 | binding | 2808/7801 |
|  | GO:0045735 | nutrient reservoir activity | 10/7801 |
|  | GO:0031386 | protein tag | 3/7801 |
|  | GO:0060089 | molecular transducer activity | 86/7801 |
|  | GO:0003824 | catalytic activity | 3085/7801 |


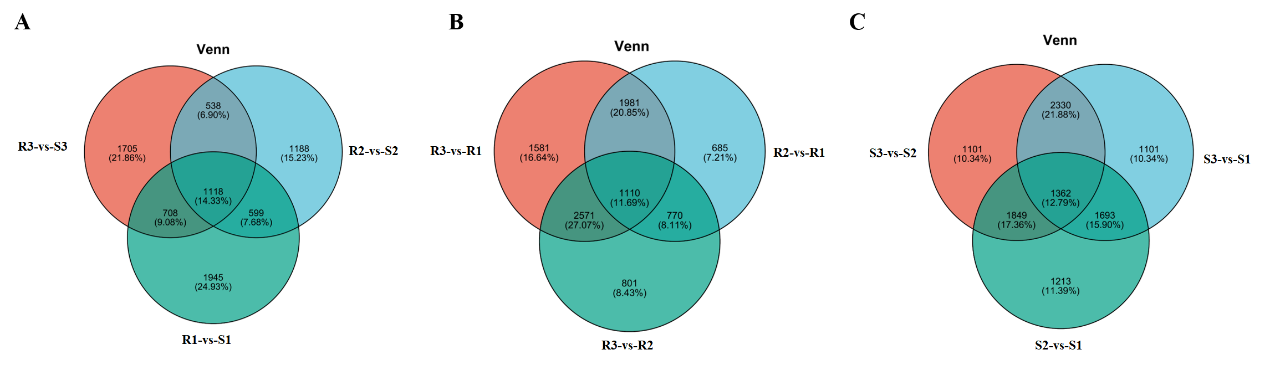
Figure S1 Venn diagram of DEGs in the samples. A: Comparative analysis of Chinese red chestnut and 'Songjiazao' at different development stages. B: Comparative analysis of Chinese red chestnut at 3 developmental stages. C: Comparative analysis of ‘Songjiazao’ at 3 developmental stages.


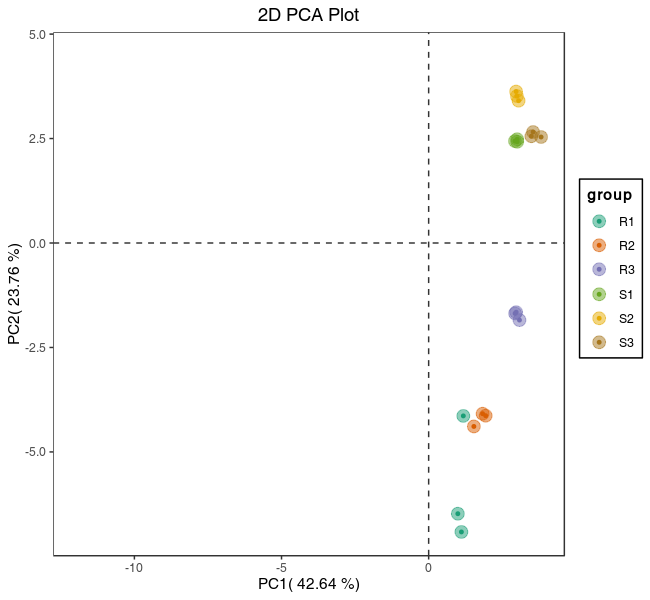


Figure S2 PCA of metabolites
